# A Demonstration of Artificial Limbs

**Published:** 1919-08-09

**Authors:** 


### A Demonstration of Artificial Limbs.

A large audience, mainly of men disabled by the loss of limbs during the war, gathered in the Central Hall, Westminster, on the last Friday in July to witness a demonstration of the use of artificial limbs. The meeting was convened by the British Red Cross and the London War Pensions Committee, presided over by Sir Laming Worthington-Evans, Minister of Pensions, in the chair. H.R.H. Prince Albert was present, and he personally inspected the exhibition of handicrafts, work of the disabled men. Addressing the audience after the inspection, the Prince said he was very pleased to meet them and to see their work. "We all know what you did in the war," he continued, "and it is up to us now to make it as easy as possible for you. It is only right that we should." Illustrations were shown by a series of cinematograph pictures of the use and proficiency of artificial limbs, and personal demonstration by two American gentlemen who had each suffered severe handicaps by loss of more than one limb, but who had overcome almost insurmountable odds by perseverance and self-help.

Judge Corley, of Texas, had lost the right arm and shoulder and left arm below the elbow in a railway accident. Without financial resources he had had to re-educate himself and learn a new way of earning a living. He studied law and politics, and was elected a county Judge, which position he had held for ten years. He demonstrated the mechanism of the hook attachment to the left elbow, which he had invented and perfected himself, by which he could do all the things that ordinary people do. He could garden, play croquet, swim, and drive an automobile. He was independent of help, and one of his greatest disadvantages was that people would insist upon helping.

Mr. Michael Dowling, President of the Olivia State Bank, Olivia, Minnesota, held the interest of his hearers by the force and energy of his address and the romance of his career. At the age of fifteen years he was frozen in a blizzard, resulting in the loss of both legs below the knee, the left arm below the elbow, the fingers of the right hand, leaving only a third of the thumb—a bit of thumb which, he explained, was worth a million dollars. A son of Irish emigrants, he was without means and without relatives. He was boarded out at public expense for two years, but he got his chance by getting two terms in a college, upon which he founded the basis of self-education and his subsequent career. He is an expert motorist, and can do everything that an ordinary person can do except tie a bow tie, although he had been practising at it for thirty years. His address was full of hope and encouragement to every man similarly handicapped, and the appreciation of the audience was shown in the burst of cheering as he retired. All the men—and there were 2,000 and more—were entertained to tea at the Central Hall after the meeting. Many thanks and appreciation were accorded to Miss Ethel Wood by the men for her personal interest in their hospitality and entertainment.

